# Impact of Z chromosome inversions on gene expression in testis and liver tissues in the zebra finch

**DOI:** 10.1111/mec.17236

**Published:** 2023-12-21

**Authors:** Heidi M. Viitaniemi, Erica H. Leder, Ondřej Kauzál, Romana Stopková, Pavel Stopka, Jan T. Lifjeld, Tomáš Albrecht

**Affiliations:** ^1^ Institute of Vertebrate Biology, Czech Academy of Sciences Brno Czech Republic; ^2^ Section of Ecology and Evolution, Department of Biology University of Turku Turku Finland; ^3^ Tjärnö Marine Laboratory, Department of Marine Sciences University of Gothenburg Strömstad Sweden; ^4^ Natural History Museum University of Oslo Oslo Norway; ^5^ Department of Zoology, Faculty of Science Charles University Prague Czech Republic

**Keywords:** chromosome inversion, liver, *Taeniopygia guttata*, testis, transcriptomics

## Abstract

Chromosomal inversions have been identified in many natural populations and can be responsible for novel traits and rapid adaptation. In zebra finch, a large region on the Z chromosome has been subject to multiple inversions, which have pleiotropic effects on multiple traits but especially on sperm phenotypes, such as midpiece and flagellum length. To understand the effect, the Z inversion has on these traits, we examined testis and liver transcriptomes of young males at different maturation times. We compared gene expression differences among three inversion karyotypes: AA, B*B* and AB*, where B* denotes the inverted regions on Z with respect to A. In testis, 794 differentially expressed genes were found and most of them were located on chromosome Z. They were functionally enriched for sperm‐related traits. We also identified clusters of co‐expressed genes that matched with the inversion‐related sperm phenotypes. In liver, there were some enriched functions and some overrepresentation on chromosome Z with similar location as in testis. In both tissues, the overrepresented genes were located near the distal end of Z but also in the middle of the chromosome. For the heterokaryotype, we observed several genes with one allele being dominantly expressed, similar to expression patterns in one or the other homokaryotype. This was confirmed with SNPs for three genes, and interestingly one gene, *DMGDH*, had allele‐specific expression originating mainly from one inversion haplotype in the testis, yet both inversion haplotypes were expressed equally in the liver. This karyotype‐specific difference in tissue‐specific expression suggests a pleiotropic effect of the inversion and thus suggests a mechanism for divergent phenotypic effects resulting from an inversion.

## 1 INTRODUCTION

Intraspecific phenotypic polymorphism observed in nature has been explained by genetic polymorphism ranging in size from single to short nucleotide polymorphisms (e.g. Chan et al., 2010) to large chromosomal rearrangements and inversions (e.g. Koch et al., 2021). Chromosomal inversions have been identified in many natural populations, and their effects on adaptation have been studied in a variety of systems (Berdan et al., 2021; Crow et al., 2020; Koch et al., 2021; Lavington & Kern, 2017; Puig et al., 2015). Depending on the location and size of the inversion, gene sequence in spanning breakpoints will be disrupted or gene regulation can be distorted if the landscape regulatory elements up‐ or downstream from the gene is altered (Puig et al., 2015). Regulation of transcription is affected by genomic architecture, chromatin landscape and cis‐regulatory regions all of which result in tissue‐specific gene expression patterns (Buchberger et al., 2019; Kleinjan & van Heyningen, 2005) and can thus be impacted by an inversion in a tissue‐specific manner. For example, an inversion may disrupt the 3D conformation involving one tissue‐specific enhancer but not that of another tissue which would be reflected in differences in tissue‐specific expression with respect to the various inversion karyotypes. Discerning how inversions contribute to evolutionary novelty thus requires an understanding of the regulatory machinery across varied tissues to investigate the pleiotropic effects of inversions.

In zebra finch (*Taeniopygia guttata*), an Australian grassfinch in the family Estrildidae, inversions on the Z chromosome seem to have a pleiotropic effect on multiple traits. As much as 90% of variance in sperm midpiece length is explained by the inversion. These inversions are also associated with various aspects of individual physiology, such as visible fat deposition rates, body mass and beak length (Knief et al., 2016). Three common segregating inversion haplotypes on the Z have been characterized from wild zebra finch populations with A presumably representing the ancestral form due to its higher heterozygosity in the wild, and B and C representing the derived inversion haplotypes (Knief et al., 2016; Pei, 2021). A fourth inversion, type D, also exists, but it is not as common as the other three (Knief et al., 2017).

The A, B and C inversions have been studied with respect to sperm morphology, and the karyotype is associated with variation in two main structures of sperm cells, the flagellum and the midpiece. Males with the AA karyotype have the longest flagellum and males with the BB karyotype have the shortest flagellum (Kim et al., 2017; Knief et al., 2017). Heterokaryotypes AB and AC have an intermediate flagellum length between AA and BB, while heterokaryotype BC and homokaryotype CC are similar in flagellum length, and are slightly longer than BB (Kim et al., 2017; Knief et al., 2017). Further differences in the karyotypes are associated with the length of the midpiece. The midpiece contains fused mitochondria and is helically wrapped around the anterior part of the flagellum. The AA karyotype has the shortest midpiece while the other karyotypes are all longer (Kim et al., 2017; Knief et al., 2017). Because of the phenotypic similarity of the sperm from B and C heterokaryotypes (Kim et al., 2017; Knief et al., 2017), they have also been referred to as B* (Knief et al., 2016, 2017). We use the same notation here.

Since the striking variation of sperm phenotypes is linked to the inversion karyotypes, we wanted to use this system for investigating genes involved in sperm morphology. Spermatozoa have evolved to be one of the most diverse cell types in the animal kingdom (Pitnick et al., 2009). High variation in sperm size is often detected across species (Kahrl et al., 2022), but also within species (Laskemoen et al., 2013) or within populations of the same species (Lifjeld et al., 2010; Míčková et al., 2023). The inversion polymorphism on the Z chromosome in zebra finch is likely maintained in the population by overdominance, with males heterozygous for the inversion (AB*) producing ‘super sperm’ (Fisher, 2017) with a proportionally large sperm mitochondrial part (midpiece) and increased mitochondrial loading (Knief et al., 2017, 2021). ‘Super sperm’ exhibits the highest velocity compared to homokaryotypes of the inversion (Kim et al., 2017; Knief et al., 2017), and heterokaryotypic males (AB*) achieve the highest fertility and the highest within‐ and extra‐pair siring success (Knief et al., 2017). Additionally, several SNPs with an important role in spermatogenesis from the inversion region were previously identified by GWAS and linked to candidate gene by examining testis gene expression between short‐ and long‐sperm individuals (Kim et al., 2017).

Although previous studies have focused on the description of the Z chromosome inversion and its effects on sperm performance, the molecular mechanisms responsible for phenotypic polymorphism in zebra finch sperm remain unclear. Once mature, individuals constantly synthesize new sperm cells, resulting in a mixture of differentially mature spermatozoa and an extremely complex transcriptomic profile in testicular tissue. This complexity can be reduced by examining sperm cells and testes transcriptomes at a uniform stage, that is, by examining the testes of males as they mature and synthesize sperm for the first time. Indeed, by sampling testes at different time points through the first wave of spermatogenesis, specific genes involved in the various phases of sperm development were discovered in mice (Laiho et al., 2013).

In this study, we set out to identify genes that are differentially expressed among the karyotypes as sperm cells are being generated in the zebra finch. Specifically, we characterized expression transcripts at four different, histologically characterized timepoints of testis development, as a proxy for the first wave of spermatogenesis, for three karyotypes AA, AB* and B*B*; (Knief et al., 2017). Additionally, we analysed gene expression in a somatic tissue, liver, as comparison with a somatic tissue may provide support for differential regulation due to inversion karyotype or allow for disentangling potential confounding effects of expression in a reproductive tissue. Our expectation was that the inversion karyotypes would result in regulatory differences as a consequence of disruption or displacement of enhancer elements and would thus be observed as differential expression between the inversion karyotypes across spermatogenesis, either in one specific timepoint or throughout all sampled timepoints. Likewise, we expected that similar expression differences related to karyotype would be identified in the liver if the two tissues shared a regulatory mutation as a result of the inversion. We found that the inversion affects gene expression in both testis and liver, and also influences which haplotype is expressed in the heterokaryotype in some genes. Our results demonstrate a potential for pleiotropic regulatory effect due to the inversion such that for some genes, the inversion has a different impact on expression in the testes compared to expression in the liver which is more complex than parallel changes in tissue‐specific expression. This highlights the need for future research to gain a deeper understanding of the regulatory landscape.

## 2 MATERIALS AND METHODS

### 2.1 Bird husbandry and sampling

Zebra finch with different inversion karyotypes were reared at the Institute of Vertebrate Biology (Studenec, Czechia). These birds originate from the domesticated ‘Krakow’ population that was generated by hybridizing between Krakow and Seewiesen populations (population 11 and 8 in Forstmeier et al. (2007)). The lines contain the homokaryotype line AA, the likely ancestral karyotype based on higher heterozygosity in wild AA birds compared to BB or CC birds (see Knief et al., 2016), and derived inversion types B and C, referred to as the homokaryotype line B*B* as described in Knief et al. (2017).

Crosses were made between individuals in order to produce AA, AB* and B*B* males and inversion genotyping for all samples was done using six tag SNPs (Knief et al., 2017). Male offspring from the crosses were sampled between 49 and 69 days of age to capture different timepoints of testis development from immature to fully mature. Birds were euthanized by using cervical dislocation, and both right testis and liver tissue were frozen in liquid nitrogen and stored at −80°C until further analysis. Left testis was fixed in Davidson's Fixative, and later prepared for histological determination of testis development. Spermatogonia and elongating spermatids were counted in five randomly chosen sections per each testicle. Next, we used a simple maturation index based on the ratio of elongating spermatids (ES) and spermatogonia (SP), that is, (ES‐SP)/(ES + SP), which scales the values between −1 and 1. Based on these histological sections, developmental stages were determined as follows: timepoint 1 = Inactive with no signs of spermatogenesis, no elongating spermatids; timepoint 2 = Accelerating I with spermatocyte division but no elongating spermatids, no spermatozoa; timepoint 3 = Accelerating II with elongating spermatids visible and maturation index −0.7 to 0.1; timepoint 4 = Active with elongating spermatids visible and maturation index 0.1–0.7. The final data set consisted of 32 birds which spanned across the testis development stage in each of the inversion karyotypes as described in Table 1. Maturation indexes for the birds are reported in Table S1.

**TABLE 1:** Final data set, across genotypes and testis development stage, consisted of 32 birds.

Karyotype	Timepoint 1: Inactive	Timepoint 2: Accelerating I	Timepoint 3: Accelerating II	Timepoint 4: Active
AA	3	3	3	3
AB*	2	3	2	3
B*B*	2	2	3	3

*Note*: B* indicates the number of derived chromosome Z inversions an individual carries. B* can be B or C inversion type.

### 2.2 RNA extraction and sequencing

RNA from testis (right) and liver tissue was extracted with a phenol‐based phase separation (Tri‐reagent, Ambion) following the Trizol‐protocol from SIGMA with minor modifications as follows: Approximately 100 mg piece of liver/whole testis was taken and 1 mL of Tri was added on the frozen tissue along with a metal bead. Tissue was homogenized using a TissueLyzer (Qiagen) for 30 s with frequency 30/s three times for the liver and one times for 30 s with frequency 30/s for testis. The bead was removed, and 100 μL of 1‐bromo‐3 chloropropane (SIGMA) was added to the sample. The sample was then shaken vigorously for 15 seconds and incubated at room temperature (RT) for 10 min and centrifuged at 4°C for 15 min at 12000*g*. The aqueous layer was carefully pipetted out to a clean tube and 500 μL of isopropanol (SIGMA) was added. Tubes were inverted a few times to allow sufficient mixing. After 10 min incubation at RT, tubes were centrifuged at 4°C for 10 min at 12000*g* to pellet the RNA. To clean the pellet, 1.5 mL of 75% EtOH was added, and the sample was left overnight at 4°C.

To ensure no traces of DNA were left in the extracted RNA, all samples were treated with DNAse (RQ1, Promega) as follows: EtOH was removed from samples after centrifuging at 4°C for 5 minutes at 7500*g*. Samples were left to dry for 5–10 minutes in RT after which 25 μL of nuclease‐free H_2_O (Ambion) was added along with 3 μL RQ1 buffer and 2.5 μL of RQ1 DNAse. Samples were mixed gently and incubated 37°C for 45 minutes after which the samples were cooled on ice. To remove traces of the enzyme and DNA, all samples were extracted a second time following the previous protocol but scaling all the reagent volumes down to 400 μL of Tri‐Reagent. RNA samples were stored in 75% EtoH at −20°C until prepared for analyses by dissolving into 17 μL of nuclease‐free H_2_O.

The RNA samples were quantified with Nanodrop and qualified with Bioanalyzer 2100 (Agilent). In total, 200 ng of RNA was used for TruSeq® Stranded mRNA (Illumina) library preparation and sequenced 100PE with two lanes of NovaSeq 6000 S1 (Illumina). Library preparation and sequencing were performed at the Finnish Functional Genomics Centre (Turku, Finland).

Reads were trimmed with cutadapt v2.7 (Martin, 2011) prior to alignment against the zebra finch genome (Ensembl bTaeGut1_v1.p, assembly GCA_003957565.2). Alignment was performed with STAR v2.7.2 (Dobin et al., 2013) using the primary assembly and the corresponding gtf annotation file (Ensembl release 100 bTaeGut1_v1.p). Read counts per gene were calculated with *quantMode* option provided in STAR.

### 2.3 Differential expression analysis

Prior to normalization and differential expression analysis, the count data were prefiltered to account for individual variation in read depths within a gene and to focus on genes where the coverage is high. In each sample, all values below 10 in the count data were transformed into 0 after which edgeR v3.40.2 (Robinson et al., 2009) *filterByExpr* was run with minimum count of 10 and total count of 320 to obtain a gene list for downstream analysis. This prefiltered gene list was then used to select genes to retain for differential expression analysis but using the original counts data (i.e. not prefiltered). Genes retained in the analysis were normalized using TMM method, and then voom (Law et al., 2014) was run to obtain mean–variance trend to add that to the differential analysis to account for additional variance in genes. Normalization was visually inspected by plotting the raw and normalized count data for testis and liver (Figures S1 and S2). All analyses were performed in R 4.2.2 (R Core Team, 2022).

**FIGURE 1:**
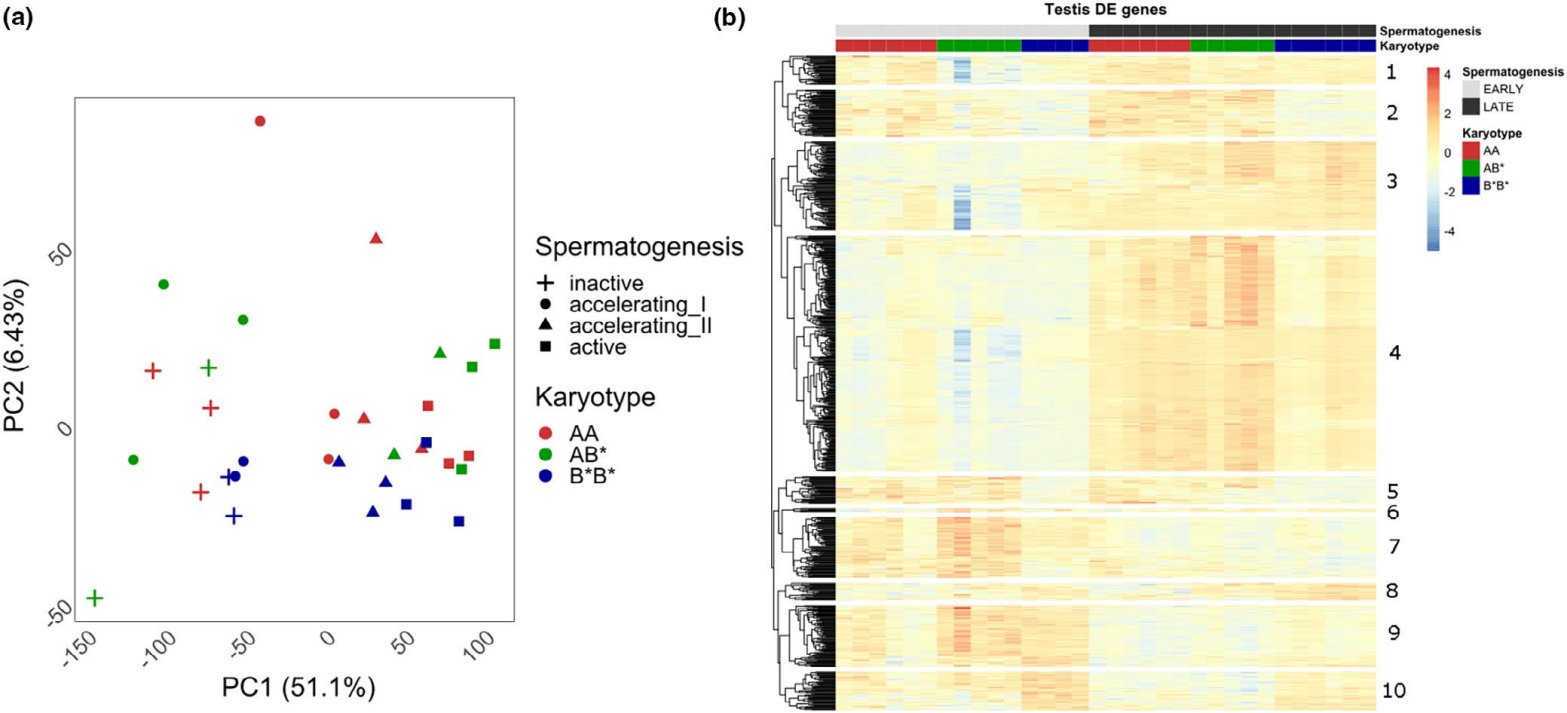
Expression in testis. (a) PCA plot of all genes expressed in testis. PC1 and PC2 explained 58% of the variation which matches progression of the developmental timepoints we sampled. Stages of spermatogenesis are indicated by shapes: plus for inactive, circle for accelerating I, triangle for accelerating II and square for active. Colour indicates the different genotypes AA = red, AB* = green and B*B* = blue. (b) Heatmap of the 794 DE genes in testis identified across the six contrasts performed in limma where significance was determined across all the contrasts with adjusted *p*‐value <.05. Colour scale is Z‐score which is calculated across all samples within each gene. Top rows indicate samples karyotype with AA = red, AB* = green and B*B* = blue and testis developmental stage with light = inactive, light grey = accelerating I, dark grey = accelerating II and black = active. Number refers to the gene cluster used in functional analysis.

Liver and testis tissue were analysed separately for differential expression. Analysis was done with limma v3.54.2 (Smyth, 2005) using *lmFit* and *eBayes*. Contrasts in limma were done using *makeContrasts*. For testis, as the sample sizes were low and the samples mostly formed two groups (Figure 1a), inactive and accelerating I samples were pooled to form group EARLY and accelerating II with active to form LATE group. All pairwise comparisons among the three inversion genotypes were performed with the two groups. For example, within EARLY group, the following contrasts were examined: AA‐AB*, AA‐B*B*, AB*‐B*B*. In the liver samples, we first examined the same contrasts as for the testis tissue, but since there was no effect of testis development time in the liver tissue (Figure S3), all timepoints within an inversion genotype were merged. Therefore, the contrast matrix was determined between inversion genotypes AA‐AB*, AA‐B*B* and AB*‐B*B* by averaging the expression for each inversion genotype across the development groups. Significance was determined separately in each tissue and across the contrast matrix (6 contrasts for testis; 3 contrasts for liver) with *decideTests* for the fits using method ‘global’ and *p*‐value .05.

To test for difference in expression level resulting from sex chromosome‐specific regulation, we calculated the ratio of median expression in Z and in autosomes (excluding scaffolds) grouped by karyotype, tissue and developmental timepoint. Expression level was determined with logCPM. To estimate bias in the number of genes expressed from the Z chromosome compared to genes expressed from autosomes Chi‐squared test with Yates continuity correction was performed in each of the contrasts on the number of observed DE genes against the number of genes in genome.

### 2.4 SNP calling along the Z chromosome

To be able to estimate allele‐specific expression from the RNAseq reads, variant calling was done in both tissues separately. Using bcftools v1.16 (Danecek et al., 2021), *mpileup* and *call* were used to call the variants from the bam files of the mapped reads specifying an output with FORMAT/DP, AD, ADF, ADR to allow to separate the allele counts. Command *filter* was used to remove low quality (QUAL < 999) loci, indels and low coverage (INFO/DP < 640) variants and then variants were filtered for individual depth of 8 (DP < 8) and for genotype quality of 10 (GQ < 10). Retained variants were mapped to genes in Z chromosome using bedtools v2.30.0 *intersectBed* command and the allele count information from the vcf files was compared across karyotypes to identify the A and B* alleles in a gene. Verification of the allele counts and calling of the A and B* alleles were manually checked to confirm haplotypes. Expression of A and B* is presented as a proportion of the allele A against all counts across all the informative SNPs in a gene.

To examine the overall SNP pattern across the Z chromosome for estimating the karyotype in our data, bam files for liver and testis were merged to increase the coverage, and variant calling was performed the same way as above for the separate tissues. Variants were filtered for individual depth of 8 (DP < 8) and for genotype quality of 10 (GQ < 10) and no missing data. This resulted in 6474 SNPs. These were used in the R package GenotypePlot v0.2.1 (Whiting, 2022) to identify the inversion pattern across the Z chromosome and to verify the karyotypes. For the genotype plotting invariant sites were not allowed, and genotypes were polarized towards the AA individuals to better visualize the pattern of homozygous and heterozygous regions among the AA, AB* and B*B* individuals. We first examined the full chromosome Z (Figure 5) and then focused on the region between 22.5 Mb until the end of the chromosome (Figure S8).

### 2.5 Functional annotation

ShinyGO v0.77 (Ge et al., 2018) was used to identify functional enrichment of the differentially expressed genes. Species was set to zebra finch and ensembl geneID was used as input identifiers. The list of the expressed genes in testis (16360) and liver (14404) was used as the background for enrichment for testis and liver respectively. For statistical enrichment in genomic locations, a window size of 4 Mb with four steps in a window were selected for testis and window size of 8 Mb with four steps in a window was chosen for liver due sparsity of genes in liver compared to testis. Significance within window was determined with FDR cut‐off of 0.01 for both tissues.

Functional overlap of GO terms between the contrasts was investigated with R package ViSEAGO v1.12.0 (Brionne et al., 2019) using the semantic clustering of enriched GO terms identified from each contrasts. To increase the functional annotation for zebra finch, a custom gene ontology database was built by merging the zebra finch GO annotation with chicken GO annotation (*Gallus gallus, bGalGal1.mat.broiler.GRCg7b*) for 1:1 orthologs (all of which were downloaded from Biomart) and then used for annotating the DE genes. All genes expressed in testis and liver were used as background, similar to ShinyGO. First all GO terms were identified for ontologies Biological Process and Cellular Component and node size of 5, and then statistical significance was determined with the *classic* algorithm and *fishers* test. Identified terms from contrasts in liver and gene clusters in testis were merged for identifying clustering in GO terms. Semantic similarity distance between the terms was calculated using the Wang method and then the Ward2 method for aggregation of the similar terms.

### 2.6 Transcription factors

For genes which were found significant in both liver and testis (49 genes), potential upstream regulators were investigated by extracting a 5‐Kb region upstream from the gene start using bedtools *getFasta* with the reference genome. For genes on the reverse strand, the sequence was reverse complemented. Sequences were input to SEA (Bailey & Grant, 2021) using a shuffled background, setting parameter ‘order to’ 2 for determining the significance and choosing right alignment for identifying the motifs from upstream sequences. For the motif database, JASPAR 2022 CORE (Castro‐Mondragon et al., 2022) for vertebrates was chosen.

## 3 RESULTS

On average, testis samples had 35,184,128 reads and liver samples had 29,790,781 reads with average mapping rates being 85.21% and 84.58% respectively (Table S1). After filtering and normalization in edgeR, reads were compared in a principal component analysis. Reads clustered based on tissue type along PC1 (Figure S3), whereas the testis reads were spread out along PC2 following the progression of the testis development. The liver reads were clustered together as one group along both axes (Figure S3). The timepoints of testis development did not seem to have an impact on liver tissue expression, even though there could potentially have been a metabolic change as the males mature. After this exploratory analysis, differential expression analysis was performed separately for both tissues.

### 3.1 Differential expression in testis

A total of 16,360 genes were expressed in the testis after removing lowly expressed genes. (Table S2). In the PCA analysis, PC1 explained 51.1% of the variation and the largest difference among adjacent timepoints was between accelerating I and accelerating II with inactive and accelerating I more similar, and accelerating II and active more similar (Figure 1a). Therefore, we merged the developmental timepoints into EARLY and LATE. The pairwise contrasts between the karyotypes in the EARLY and LATE identified 794 genes as differentially expressed (DE) among karyotypes in one or more contrasts (Table S2; Figure S4). Clustering of the differentially expressed genes based on logCPM indicates that there is a clear division between EARLY and LATE development stages along with 10 distinct cluster patterns in expression levels (Figure 1b; Table S2).

There were 259 significant GO terms for biological process and cellular component identified from the functional enrichment analysis of the 10 cluster in the DE genes. After semantic clustering, 41 semantic GO term clusters remained. These were separating out the 10 gene clusters functionally (Table S4). Cluster 4 was the largest (Figure 1b) and it has the most relevant GO terms for sperm development within this cluster: for example, GO:0036126 sperm flagellum, GO:0007286 spermatid development (Table S4). Additionally, cluster 4 also has enrichment for many GO terms involved with RNA transcription and processing (Table S4), likely highlighting the high transcriptional activity involved in spermatogenesis.

Statistically significant enrichment of DE genes based on genomic location was found on chromosome Z (Figure S5). One region was located close to the end of the chromosome Z between 64.0 Mb and 68.8 Mb. This area contains nine protein coding genes and two lncRNA genes (Table S5). A second region with enrichment of differentially expressed genes is located before the centromere region in chromosome Z between 22.7 and 27.9 Mb, and it contains nine protein coding genes and eight lncRNA genes (Table S5).

### 3.2 Differential expression in liver

**FIGURE 2:**
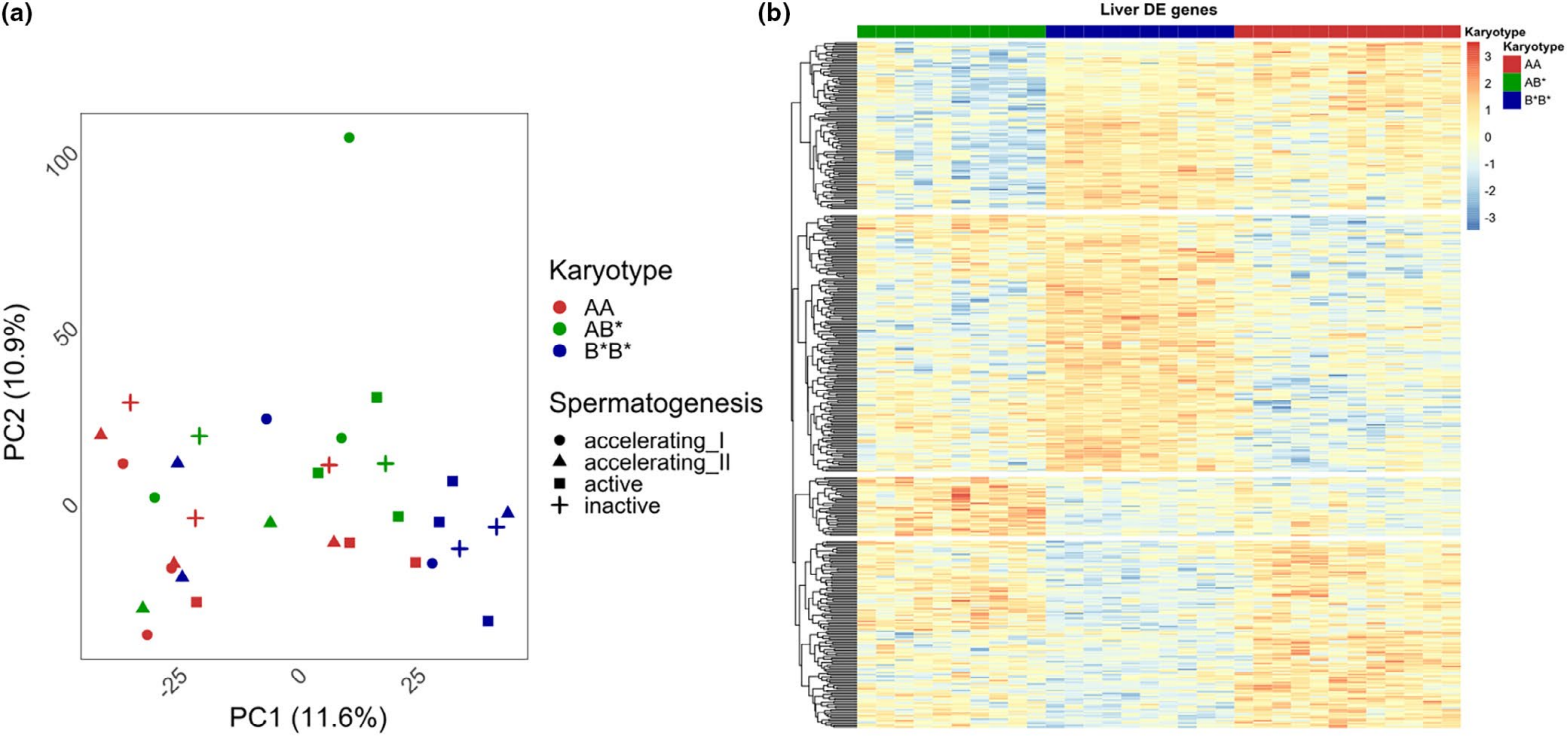
PCA plot of liver genes. (a) PC analysis was done for all expressed genes in liver. PC1 and PC2 explained 22% of the variation. In liver, the developmental timepoints did not affect sample distribution. Stages of spermatogenesis are indicated by shapes: plus for inactive, circle for accelerating I, triangle for accelerating II and square for active. Colour indicates the different genotypes AA = red, AB* = green and B*B* = blue. (b) Heatmap of the 420 DE genes in liver identified across the three contrasts ran in limma where significance was determined across all the contrasts with adjusted *p*‐value <.05. Colour scale is Z‐score which is calculated across all samples by gene. Top rows indicate samples karyotype with AA = red, AB* = green and B*B* = blue and testis developmental stage with light = inactive, light grey = accelerating I, dark grey = accelerating II and black = active.

A total of 14,404 genes were identified from liver (Table S3) with 420 genes differentially expressed among the karyotypes. In general, liver was not clearly separated by testis development stage but rather by karyotype, and the first PC explained 11% of the variation among the expressed genes (Figure 2). In liver, the same pattern as in testis of B*B* being different from AA and AB* was also seen and there was overlap between the DE genes for contrasts AA‐B*B* and AB*‐B*B* (Table S3; Figure S4B). In total, 92 GO terms were identified among the DE genes among karyotype comparisons. The semantic clustering of GO terms identified in total 26 clusters which were characterized by small overlap between the contrasts (Table S6). Functions were related to, for example, regulation of metabolic processes, fatty acid metabolism and regulation and development of tissues and cells (Table S6). For the comparison between AA‐B*B*, we identified two genomic locations where there was an enrichment of DE genes compared to the background of expressed genes (Figure S6). These were located on chromosome Z, and one matched to distal location identified in testis at 64.1–69.0 Mb with three protein coding genes and three lncRNA genes (Table S5; Figure S5). At 33.5–39.9 Mb where there were four protein coding genes and three lncRNA genes (Table S5).

### 3.3 Karyotype‐specific expression patterns in the testis DE genes

**FIGURE 3:**
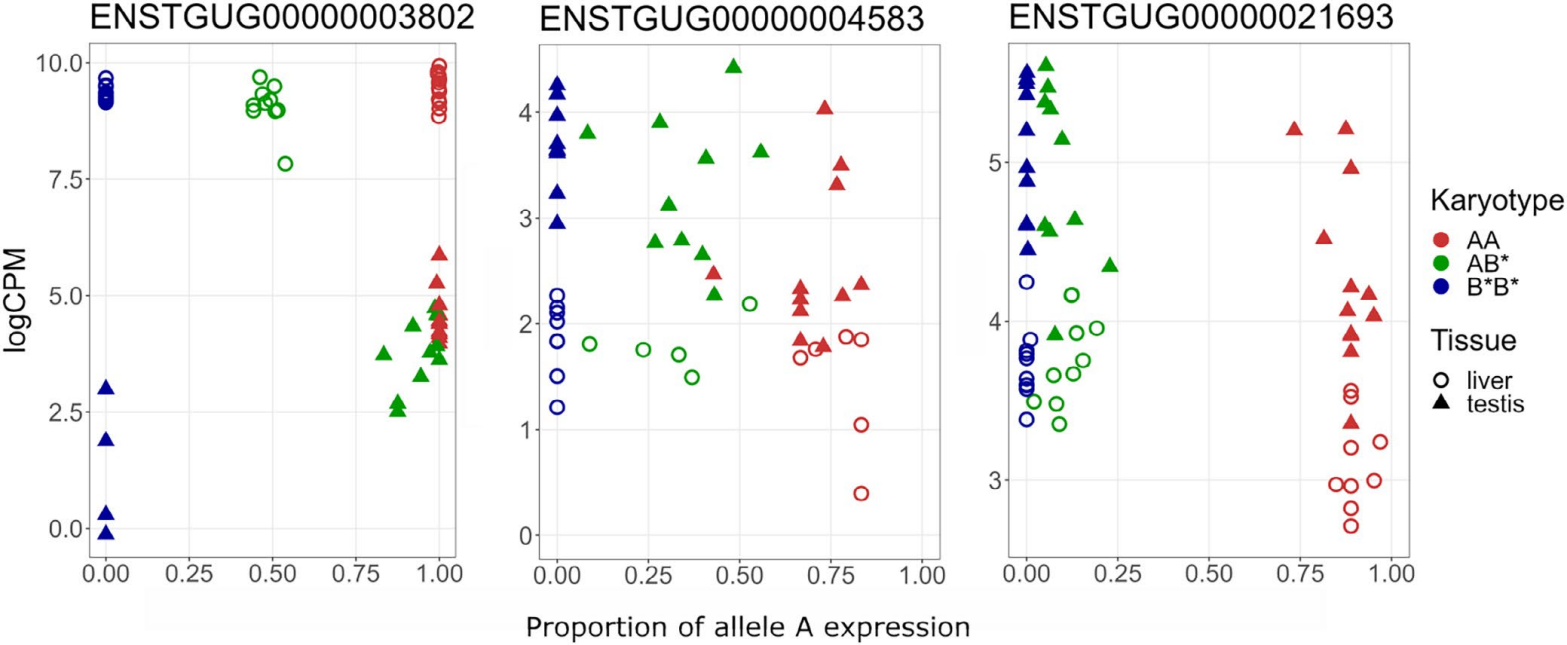
Inversion‐specific expression for ENSTGUG00000021152 (lncRNA), ENSTGUG00000021693 (novel gene), DMGDH (ENSTGUG00000003802), ENSTGUG00000020830 (lncRNA), ENSTGUG00000006415 (protein coding), ENSTGUG00000020596 (lncRNA) and FREM1 (ENSTGUG00000004583) which are located on chromosome Z and ENSTGUG00000020011 (lncRNA) and ENSTGUG00000021745 (lncRNA) located on chromosome 2 and BIN1 (ENSTGUG00000004882) on chromosome 7. For each developmental stage within each karyotype, the mean expression in logCPM is represented and error bars indicate the minimum and maximum value in the group. Karyotype is indicated with AA = red, AB* = green and B*B* = blue.

When looking at the DE gene expression pattern across testis development, the heterozygote AB* occasionally had more similar expression to one of the homokaryotypes compared to having intermediate level of expression (Figure 3). A pattern where the heterozygote AB* was biased towards the B* inversion‐specific in expression level in across all testis development stages was seen in, for example, ENSTGUG00000021152 (lncRNA) and ENSTGUG00000021693 (novel gene) which are located on chromosome Z and ENSTGUG00000020011 (lncRNA) and ENSTGUG00000021745 (lncRNA) located on chromosome 2 and BIN1 (ENSTGUG00000004882) on chromosome 7. Gene *DMGDH* (ENSTGUG00000003802) located on chromosome Z was biased towards the A inversion‐specific expression level in the heterozygote. A third type of pattern, where the heterozygote goes from intermediate expression level to either A or B* inversion‐specific expression, was observed in ENSTGUG00000020830 (lncRNA) and *FREM1* (ENSTGUG00000004583) which are also located on Z. Fourth pattern type was where expression in heterokaryotype AB* changed from one homokaryotype to the other between the EARLY and LATE division as seen in genes ENSTGUG00000006415 (protein coding) and ENSTGUG00000020596 (lncRNA), both of which are located in chromosome Z.

### 3.4 Effect of karyotype between tissues

**FIGURE 4:**
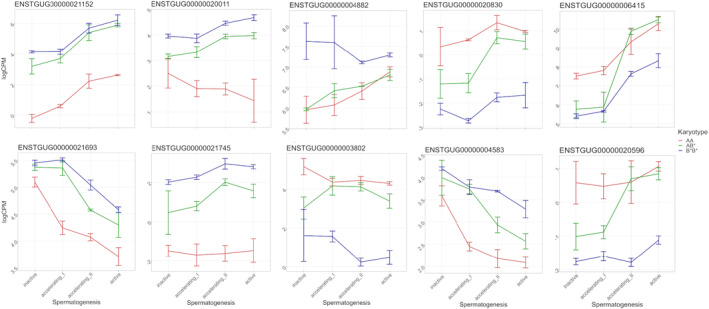
Expression compared with allele frequency in testis and liver for genes DMGDH (ENSTGUG00000003802), FREM1 (ENSTGUG00000004583) and ENSTGUG00000021693. Allele frequency on the x‐axis indicates the proportion of the A inversion‐specific variant counts against total depth across all SNP in gene. Counts for a gene were obtained from variants of the reads mapped to the gene. Expression level is represented by logCPM on y‐axis. Each symbol represents one individual. Liver = circle and testis = triangle, and colour indicates the different genotypes AA = red, AB = green and BB = blue.

To further examine the observed patterns in allele‐specific expression in heterokaryotypes, variant calling was performed on the significant genes on the Z chromosome. Allele count information was used to measure proportion of A allele expression across the gene, since it is assumed that the reference genome individual was BB, although there is a chance of it being BC (Itoh et al., 2011; Knief et al., 2016). For *DMGDH*, allele‐specific expression in the heterozygote AB* was found in testis, but not in liver tissue (Figure 4). For gene ENSTGUG00000021693, allele‐specific expression was observed in both tissue types in the heterozygote AB* (Figure 4). For *FREM1*, expression of both karyotype‐specific alleles was observed in the heterozygote AB* (Figure 4). For the remaining five out of the eight genes, no SNPs passing our filtering were located in the gene, and allele‐specific expression from a specific karyotype could not be verified. For other expressed genes, the patterns for allele‐specific expression were not determined as clearly since not all variants within a gene could be reliably assigned to either A or B* inversion with our data due to lack of fully fixed allele variants between AA and B*B*.

### 3.5 Effect of inversion on expression from Z and autosomes

In testis, in the EARLY contrast AA‐AB* (*p*‐value <.05, *X* ^2^ = 12.543) and AB*‐B*B* (*p*‐value <.05, *X* ^2^ = 73.535) as well as in LATE contrasts AB*‐B*B* (*p*‐value <.05, *X* ^2^ = 9.601) and AA‐B*B* (*p*‐value <.05, *X* ^2^ = 40.967), DE genes were more likely to be in Z than in autosomes when comparing to the genomic distribution of genes as the expectation (Table S7). In liver, only contrast AA to B*B* (*p*‐value <.05, *X* ^2^ = 37.774) had significantly more genes on Z than on autosomes (Table S7). Z versus autosome expression level between karyotypes and tissues was equal. Only in liver, the Z:autosome ratio is lower for AB* than in AA or B*B*. Thus, our data did not indicate a bias in expression level between genes expressed from Z versus autosomes (Table S8; Figure S7). This holds true across karyotypes, tissues and early and late spermatogenesis.

### 3.6 Chromosome Z inversion landscape in bTaeGut1_v1.p

**FIGURE 5:**
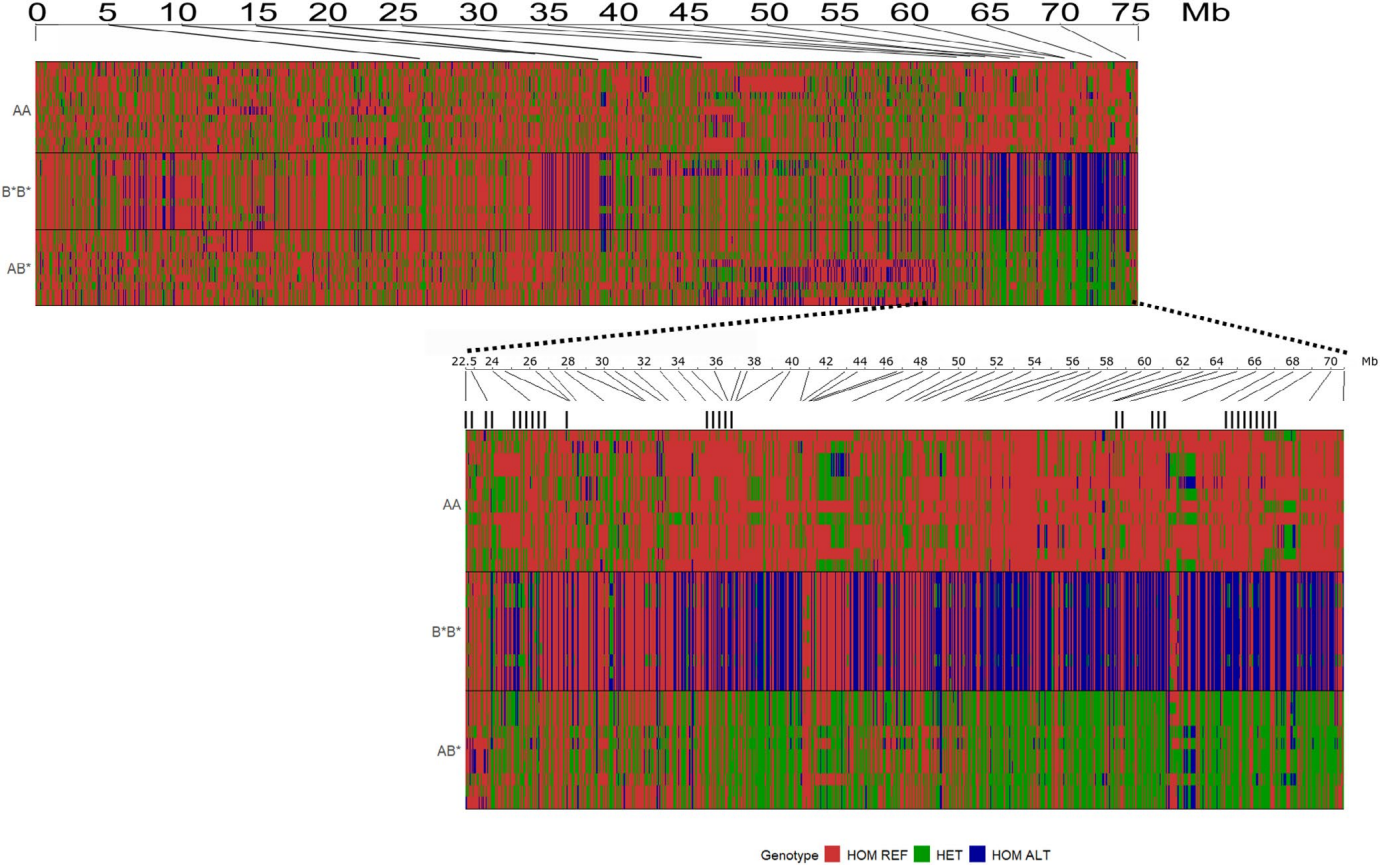
Genotype plot from 6371 SNPs from start until the end of the chromosome Z with closeup to region from 22.5 to 72 Mb with 1061 SNPs. The SNPs were acquired after merging the liver and testis transcriptomes. Samples are represented vertically, and the physical location along chromosome Z is at the top. The downward lines indicate the chromosome position among the SNPs as the SNP density along the Z is not constant. Each SNP genotype homozygous for the tagut1.1 reference is represented by red, homozygous for the alternative allele is in blue and the heterozygote is in green. Black lines in the closeup indicate the regions where overrepresentation of DE genes was identified in both tissues.

In an attempt to place our genes in relation to putative breakpoints, we examined SNPs from our transcriptomes for the karyotypes across Z. We merged our transcriptome SNP data from both tissues for each individual and proceeded with SNP calling as above. In general, there are much fewer SNPs in genes which creates some difficulty with detecting specific breakpoints; therefore, we attempted to compare our SNPs with previously published SNP and karyotype data for the Z chromosome from Kim et al. (2018). In general, our SNPs matched the Kim data after reanalysing the Kim data to reflect the newer reference assembly in bTaeGut1_v1.p (Data S2, Figure S9). The region from 25 Mb to 70 Mb at the distal end of Z discriminates our AA and B*B* from each other and corresponds to the data of Kim et al. (2018). There are some regions, however, that seem to be distinct to a few individuals (Figure 5). However, these are likely representing familial relationships as several individuals are brothers. Specifically, there are nine families from which 27 of our 32 samples originate from (Table S1). This is especially seen in B*B* where only two families produced the eight samples.

Also, we cannot completely neglect the effect of allele‐specific expression to our variant calls, particularly in the case of AB* individuals. However, using both our data and that of Kim et al. (2018), we can estimate the positions of the breakpoints from the genotypes (Figure S8) and pairwise LD patterns (Figure S9), and especially visualize large blocks of fixed differences between the homokaryotypes which may impact gene expression. One region from 7 Mb extending until 17 Mb discriminates A and B, whereas from 17 MB to 22 Mb is a region with large homozygosity between A and B but some C specific regions are located here. From 22 Mb until the end of the chromosome A, B and C homokaryotypes and heterokaryotypes are discriminated from each other more clearly. We hypothesize that these boundaries indicate the inversion breakpoints. Thus, the potential inversion regions are plausible for where we see overrepresentation of differentially expressed gene in testis and liver at 22.5–27.9 Mb, 33.5–39.9 Mb and 64.0–68.9 Mb (Figure 5).

### 3.7 Transcription factor motifs

In total, 49 genes were identified as differentially expressed in both tissues, and the direction of expression between the karyotypes was similar across tissues (Table S9). Upstream motif analysis identified 52 putative‐binding motifs for these genes (Table S10). All transcription factor genes associated with these motifs had an ortholog in the zebra finch. Of the identified transcription factors, 21 were found expressed in both tissues, 12 were only expressed in testis (Table S10).

## 4 DISCUSSION

As the various inversion karyotypes on the Z chromosome involve many genes, it is not surprising that the effects of the inversions on gene expression are not just restricted to the testis but are also observed in liver. Our most striking finding was that some genes show an expression pattern more similar to one of the karyotypes when the individual is heterokaryotypic rather than an intermediate pattern of expression during the ontogeny of spermatogenesis (Figure 3). Interestingly, this is not only towards one inversion karyotype but changes in a gene‐specific manner. This karyotypic expression pattern also occurs for some genes that are not on the Z chromosome which suggests a trans‐regulatory role, possibly from one of the many lncRNAs on the Z chromosome, some of which also have allele‐specific expression by karyotype (Figure 4). Additionally, it seems that one gene (*DMGDH*) has transcripts that are only expressed from one karyotype in the testis, but transcripts from both karyotypes are expressed in the liver in the heterokaryotypic individuals. Since the heterokaryotype in liver has equal numbers of A and B* alleles expressed, and has the same expression in all karyotypes, this effect is likely the result of a testes tissue‐specific enhancer being altered in the B* haplotype. This could also be the result of some type of regulatory interaction between the A and the B* haplotypes since DMGDH is expressed in B*B*, but to a lesser extent than AA and AB*, and in the heterokaryotype, there are very few B* alleles produced among the AB* individuals.

### 4.1 Patterns of transcript expression along the Z chromosome

When comparing the locations of the differentially expressed genes and genotypes called from the transcriptomes to the pattern of LD and genotypes along the Z chromosome using SNP data from Kim et al. (2018), it is plausible that the inversions contribute to the expression patterns observed between the AA, B*B* and AB* karyotypes through differences in physical location or SNP variation in regulatory motifs of genes. Furthermore, our transcriptome data together with SNP data from Kim et al. (2018) show that instead of having one large 56 Mb inversion covering most of the chromosome as seen with the taeGut3.2.4 assembly, the chromosome Z landscape may have several inverted regions (Pei, 2021). In general, inversions can affect gene expression in multiple ways: for example, disrupting the gene sequence, distorting gene regulation through the 3D regulatory conformation or altering chromatin landscape (Puig et al., 2015). Distortion of gene regulation is the most plausible explanation for the expression patterns observed (Figures 3 and 4) since if a gene is disrupted, it is likely non‐functional. Pleiotropic effects across tissues could be explained by regulators, such as transcription factors or enhancers. Although we did not identify obvious candidate transcription factors from Z chromosome as upstream regulators for the 49 genes differentially expressed in both tissues, it is likely that enhancer sites are more impacted from inversions than transcription binding sites since enhancers can be very distant from the gene and are known to be important for temporal and tissue‐specific gene regulation (Kosuthova & Solc, 2022; Sabarís et al., 2019). Unfortunately, the techniques required to examine enhancers are not possible within the scope of this study, and we cannot address the 3D structure and the regulatory landscape at this time (Kleinjan & van Heyningen, 2005). Nevertheless, investigating effects of inversion on gene expression across tissues, such as done in this study, provides further resolution to start resolving questions about the role of *cis*‐ and *trans*‐regulation and epigenetic regulation and how regulatory elements are affected by inversions changing the structure of chromatin (Crow et al., 2020; Wellenreuther et al., 2019).

Given that many long‐non‐coding RNA genes were also differentially expressed due to the inversion suggests that they affect gene regulation in a tissue or cell‐specific manner, as seen in humans (Zhang et al., 2019), or affect the epigenetic pattern of gene regulation (Fatica & Bozzoni, 2014; Wei et al., 2017). Epigenetic regulation is also a potentially important mechanism for differential gene expression. In humans, gene expression in inverted regions is affected by inversion‐allele specific methylation which also showed tissue‐specific effects (Carreras‐Gallo et al., 2022). Similarly, in white‐throated sparrows, inversion ZAL2m showed allele‐specific differential methylation in a cis‐regulatory element of the vasoactive intestinal peptide gene which was previously shown to be differentially expressed between individuals carrying different inversion alleles (Prichard et al., 2022). As we are focusing on sex chromosomes, silencing mechanisms could also affect gene expression levels differently, especially between tissues. Meiotic silencing of unsynapsed chromatin (MSUC) in heterogametic sex chromosomes in developing sperm is known to happen in mammals, known as meiotic sex chromosome inactivation, MSCI (Van Der Heijden et al., 2011), but evidence for MSUC in birds remains unresolved (Pigozzi, 2022). Meiotic silencing affects primarily heterogametic individuals which are males in XY system but females in ZW systems. Also, evidence for MSCI in birds remains scarce (Guioli et al., 2012; Segami et al., 2022). As we observed no difference in Z:autosome ratio between tissues and karyotypes across the developmental timepoints (Figure S7; Table S8), we consider the MSCI not globally affecting our DE results. In further support of the lack of MSCI in birds single‐cell RNAseq in flycatchers found no support for MSCI since there was no evidence of silencing in sperm compared to other tissues (Segami et al., 2022).

Thus, the allele‐specific expression observed in *DMGDH* may be due to tissue‐specific enhancers having mutated between the A and B* haplotypes after the inversion. The other plausible reason is that the inversion distorted only one of the tissue‐specific enhancers as the gene is in the vicinity of the most distal putative breakpoint between A and the derived B and C haplotypes. *FREM1* and lncRNA ENSTGUG00000021693 showed different patterns of allele‐specific expression between tissues compared to *DMGDH*: *FREM1* is expressed at intermediate level compared to either of the homokaryotypes (AA or B*B*) and for ENSTGUG00000021693, the heterokaryotype shows uniformly B*B*‐like expression pattern. Similar patterns of inversion‐specific expression have been observed in seaweed flies (*Coelopa frigida*). The inversion Cf‐*Inv(1)* resulted in differential gene expression between the karyotypes across life stage and sex: both *cis*‐ and *trans*‐regulatory effects were identified, and they differed between the sexes and life stages (Berdan et al., 2021). The Cf‐*Inv(1)* inversion is similar to the zebra finch Z inversion as it covers most of the chromosome, but the inversion in seaweed fly is autosomal. Another feature in common with our data and other studies is that the effect of inversion on gene expression is genome‐wide and not just restricted to the chromosome containing the inversion demonstrating *trans*‐regulatory affects (Berdan et al., 2021; Crow et al., 2020; Fuller et al., 2016; Lavington & Kern, 2017).

### 4.2 Patterns between karyotypes and tissues

In testis, the DE genes were involved with functions related to cilium assembly and organization (GO:0060271, GO:0044782) (Table S4), and genes involved in these GO terms have also been identified to be involved in the human sperm microtubulome (Jumeau et al., 2017). Specifically, clusters 4 and 7 from the heatmap had enrichment for spermatid development (GO:0007286) and spermatid differentiation (GO:0048515), and cluster 4 also had enrichment for cilium (GO:0005929) and sperm flagellum (GO:0036126). Cluster 4 also is a strong candidate cluster for identifying the genes related to the flagellum length differences among karyotypes, since in the LATE group, the expression levels of the B*B* karyotype are lower than AA and AB* which correlates with the B*B* having the shortest flagellum.

Most of the DE genes in testis were from the AA and AB* comparison in the EARLY group and between AB* and B*B* in the LATE group (Table S7). Although we used histology to characterize the samples, it could be possible that expression profiles, especially in the early stage, could differ without a noticeable difference in cell types. The AA karyotype seemed to have more advanced expression profile, while AB* was more similar pattern to the inactive stages. Alternatively, these could be real effects of expression differences where AA has a suite of spermatogenesis genes that start increasing sooner than in the other karyotypes. The longer flagellum length in AA could thus be a result of an extended duration of expression rather than higher expression at a specific time.

From the viewpoint of progression of onset of spermatogenesis in our sampling design, the first two timepoints mainly contain early sperm cell stages whereas the active stage contains cell types from all of the previous stages as well (Aire, 2014), although genes seen for the first time in active stage should be involved in the final stages of sperm cell maturation. Although our analysis does not have the resolution of a single‐cell analysis, we can discriminate differences in expression which likely reflect the different developmental status of cells along spermatogenesis in the inversion karyotypes. This is also indicated in the heatmap (Figure 1b) where testis samples form a continuum of changing gene expression across the sampled timepoints from inactive to active in contrast to the pattern in the liver (Figure 2b).

### 4.3 Candidate genes

Kim et al. (2017) found eight genes in GWAS from the Z inversion associated with sperm morphology: *GADD45G*, *LRRC2*, *C9orf3*, *FBXL17* (associated with midpiece length), *DMRT2*, *LINGO2*, *ZNF462* and *RAD23B*. None of these eight genes were differentially expressed in our testis data or in liver. In the same study, Kim et al. (2017) identified over 100 DE genes between long (AA, AB or AC karyotypes) and short (BB karyotype) sperm phenotypes using a microarray, but of these, only 18 were differentially expressed in our testis transcripts and 17 of those were upregulated in long sperm phenotype individuals. Our final timepoint (active) is most comparable to other studies which are looking at mature testis where spermatogenesis is ongoing and thus contains a mixture of cells at different developmental stages (Aire, 2014).

Although we did not have large overlap between the DE genes identified by Kim et al. (2017), they found *DMGDH* as the most differentially expressed gene between the long (AA, AB or AC karyotypes) and short (BB karyotype) sperm phenotypes sampled from adult males. Our results corroborate this finding although *DMGDH* was not the most strongly differentially expressed gene in our analysis of testis. In the active stage, which would match Kim et al. (2017), the expression difference between AA and AB* was not very large (log2 FC 0.82, Table S2) whereas to B*B* the difference was much larger (log2 FC 3.9, Table S2). GO terms associated with *DMGDH* contain GO:0042426 choline catabolic process and GO:0047865 dimethylglycine dehydrogenase activity indicating that if *DMGDH* is important for sperm function it is likely through the choline pathway. In a human study, a variant in the choline dehydrogenase, *CHDH*, leads to decreased expression which affected sperm function and mobility through reduced ATP concentration (Johnson et al., 2012). So, there may be a functional link between increased *DMGDH* expression and increased sperm motility in AB* karyotype individuals through the choline metabolism pathway. Given that *DMGDH* gene expression in liver is stably higher than in testis (Figure 3), the true involvement of *DMGDH* for ‘super sperm’ in zebra finch remains unresolved.

Our data provide verification to the previous study (Kim et al., 2017) but also provide novel aspects to tissue‐specific effects of the zebra finch Z chromosome inversions. Due to the focus on testis development, we also provide insight into the potential mechanism behind the variation in sperm morphology among karyotypes. However, our data contain some limitations due to the difficulty identifying the spermatogenesis stage even with histology. Since calendar age is not an accurate indicator of spermatogenesis stage in zebra finches, we had to sample and process histology for many birds, and we still had difficulty obtaining some samples. This resulted in small sample sizes in the inactive and accelerating I stages for some karyotypes which we have tried to address by pooling the sampled developmental stage to EARLY and LATE. Another confounding factor is identifying the Z chromosome inversion breakpoints. Our SNP data from the transcriptomes provide some resolution for identifying distinct patterns of homozygosity/heterozygosity along the Z chromosome, but the density of SNPs and the read depth within the transcriptomes is limited. Since we were unable to unequivocally distinguish between the AB or AC heterokaryotype in our data, we retain caution in our interpretation and retain a similar strategy to that of Knief et al. (2017) such that the main comparison with the heterokaryotypes is the effect of carrying one ancestral and one derived inversion in comparison to homozygotes. Future exploration of the effects of inversions shaping the regulatory landscape and gene expression patterns in hetereokaryotypes would require characterizing the sequence and gene order of the different inversion types A, B, C and D, preferably in females where there is no need to phase the haplotypes.

### 4.4 Conclusion

In conclusion, our unique sampling of zebra finch Z chromosome inversion karyotypes in two different tissues across testis development revealed karyotype‐specific and tissue‐specific expression differences suggesting pleiotropic regulatory effects due to the inversion with both *cis*‐ and *trans*‐effects. The gene enrichment coincides with gene functions that would be strong candidates for the phenotypic differences observed in the sperm, that is, flagellum and midpiece length. Additionally, we identify several hundred differentially expressed genes in the liver resulting from the various inversion karyotypes. These genes may be influencing some of the physiological phenotypes previously observed such as fat deposition rates and body mass (Knief et al., 2016) since some of the enriched functions from the semantic similarity clustering are related to regulation of metabolic processes and fatty acid metabolism. Further investigation into specific karyotypes should allow for a better understanding of how inversions, by disrupting the existing regulatory landscape, can promote evolutionary novelty.

## AUTHOR CONTRIBUTIONS

Tomáš Albrecht, Erica H. Leder, Jan T. Lifjeld designed the original research plan. Ondřej Kauzál and Tomáš Albrecht maintained and sampled the birds used for the experiment at the Institute of Vertebrate Biology, Czech Republic. Romana Stopková and Pavel Stopka did the histological analysis. Heidi M. Viitaniemi designed and conducted the laboratory work, Heidi M. Viitaniemi analysed the data and wrote the manuscript together with Erica H. Leder. All authors contributed critically to the drafts and gave final approval for publication.

## FUNDING INFORMATION

This study was funded by the Czech Science Foundation (GAČR) project 19‐22538S to TA, and by the Research Council of Norway grant #301592 to JL.

## Supporting information


Data S1.

Data S2.

Figure S1.
Figure S2.
Figure S3.
Figure S4.
Figure S5.
Figure S6.
Figure S7.
Figure S8.
Figure S9.

Table S1.
Table S2.
Table S3.
Table S4.
Table S5.
Table S6.
Table S7.
Table S8.
Table S9.
Table S10.

## Data Availability

Sequences, including metadata, produced in this study have been deposited on DataDryad doi: https://doi.org/10.5061/dryad.6hdr7sr6k. All code used in the analysis is available as a supplementary. Benefits from this research accrue from the sharing of our data and results on public databases as described above.
