# Supplementary material for: Impact of Z chromosome inversions on gene expression in testis and liver tissues in the zebra finch
Source: Mol Ecol. 2023 Dec 21;33(24):e17236. doi: 10.1111/mec.17236 (PMC11628666; doi:10.1111/mec.17236)
Supplement: Supplementary file 2 — Data S2. [file MEC-33-e17236-s003.pdf]

Patterns of Z chromosome genotypes and LD in Kim at el (2017) in bTaeGut1_v1.p assembly

Previous studies on the zebra finch Z chromosome inversion (Kim et al., 2017; Knief et al., 2016, 2017) used assembly (WUSTL 3.2.4 / taeGut3.2.4). The newer bTaeGut1_v1.p assembly is more contiguous and corrects some misassemblies, but several large segments along the Z, and likely on other chromosomes, are inverted or rearranged. In order to compare the previous information from (Kim et al., 2017), we converted the SNP positions in their PLINK file (Kim *et al.*, 2018) to positions on the new genome for the Z chromosome. This was done by creating a bed file of +/- 100 bases on either side of the SNP position from the plink file, and extracting these 200 bases as a fasta file from the old assembly using bedtools (v 2.30.0) (Quinlan & Hall, 2010) *getfasta* These 200 base fragments were then mapped to the new assembly using minimap2 (v 2.24) (Li, 2018). Based on the mapping coordinates of the 200 base fragment in the new genome, the SNP position was calculated. SNPs which we couldn't map uniquely to the bTaeGut1_v1.p assembly were not included in the following analysis. The old position was replaced with the new position in vcf file output from plink by using the SNP identifier in the replacement. GenotypePlot v0.2.1 (Whiting, 2022) R package was used to plot the rematched genotypes to verify the inversion patterns between the datasets. For the genotype plotting, missing data and invariant sites were not allowed, and samples were clustered based on the provided genotypes. Rematched SNP data from (Kim et al., 2017) was also used to call all the pairwise LD pattern using PLINK v1.9. (Purcell et al., 2007) to call the r^2 while filtering for --maf 0.25, --geno 0.1 and --mind 0.5. LD was visualized with R package LDheatmap (Shin et al., 2006).
