# Supplementary material for: Impact of Z chromosome inversions on gene expression in testis and liver tissues in the zebra finch
Source: Mol Ecol. 2023 Dec 21;33(24):e17236. doi: 10.1111/mec.17236 (PMC11628666; doi:10.1111/mec.17236)
Supplement: Supplementary file 3 — Figure S1. Figure S2. Figure S3. Figure S4. Figure S5. Figure S6. Figure S7. Figure S8. Figure S9. [file MEC-33-e17236-s004.docx]

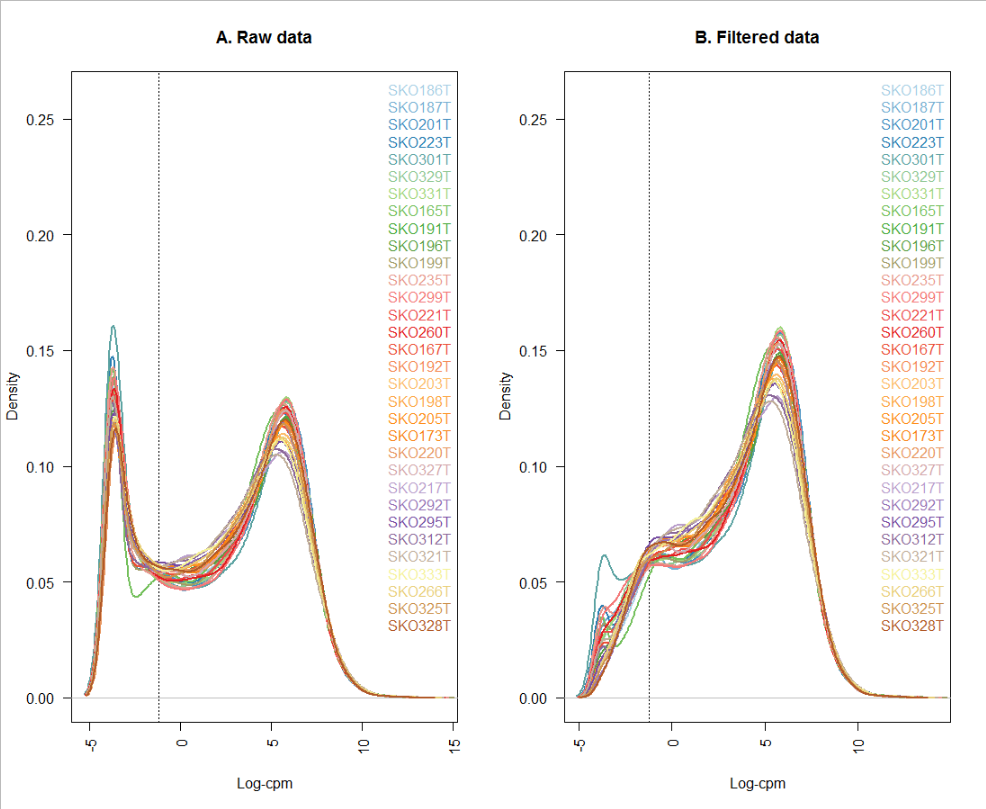


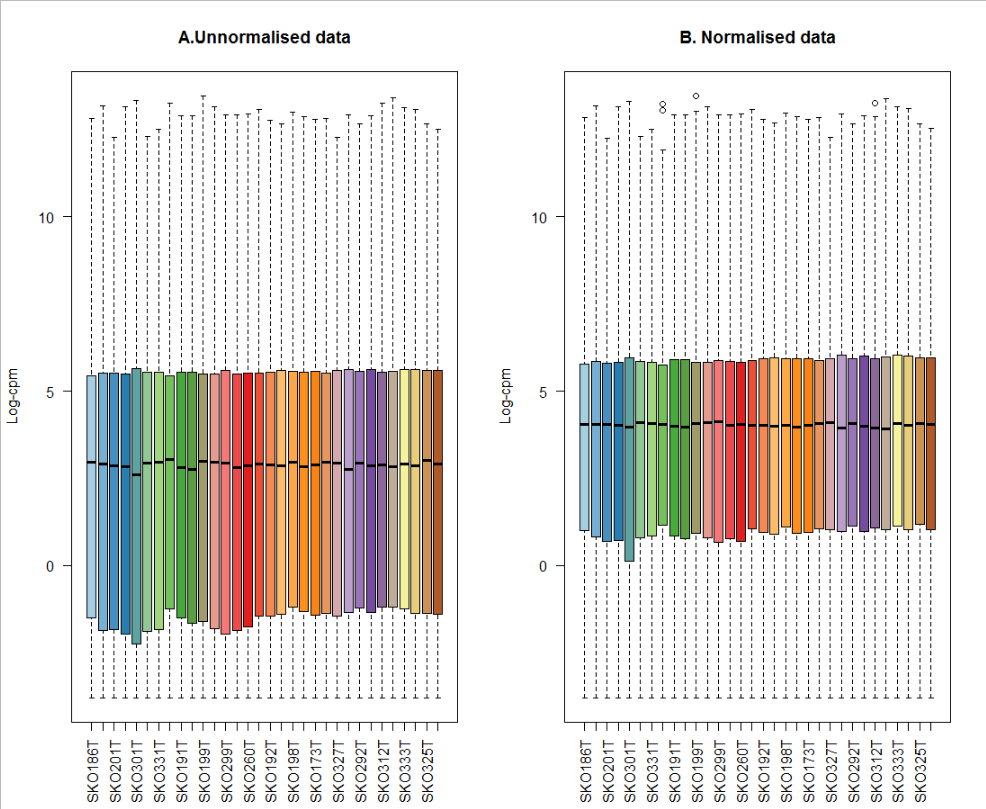


Figure S1. Effect of normalisation on the testis data.


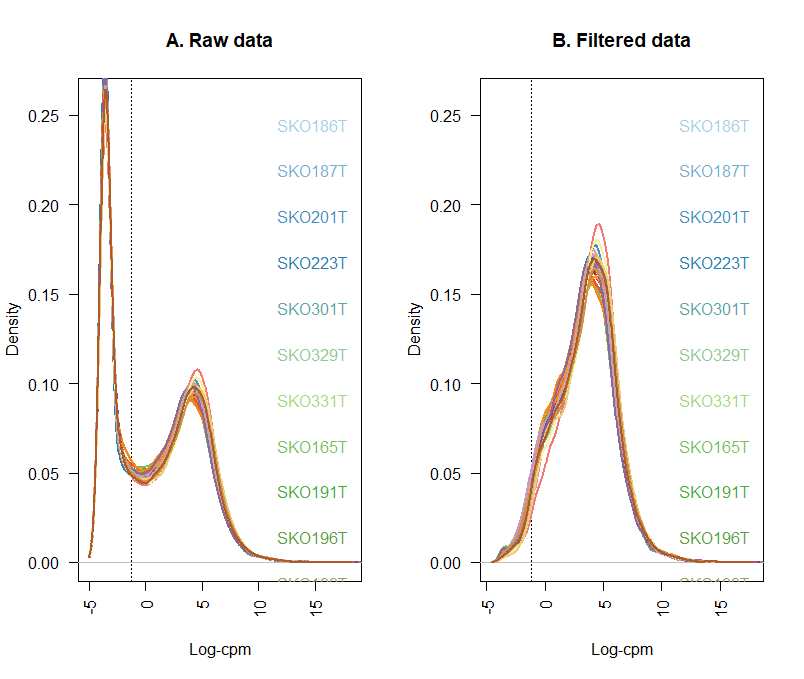


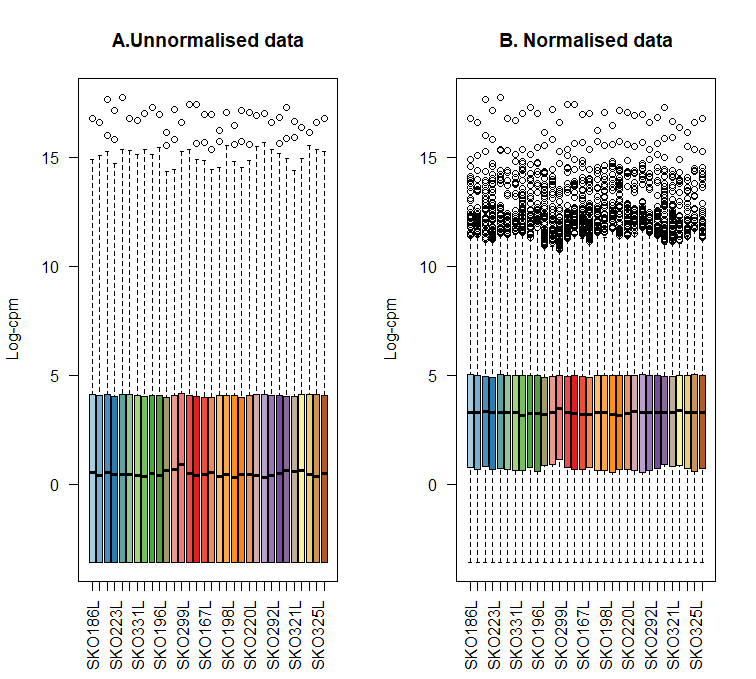


Figure S2. Effect of normalisation on the liver data.


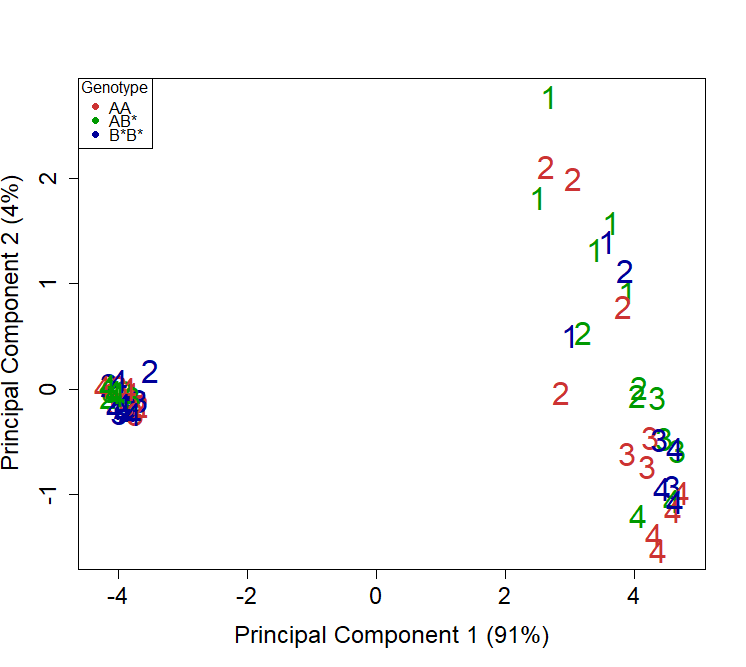


Figure S3. MDS plot of the expression using top 1000 genes for liver (L) and testis (T) as preliminary verification on the effects of the sampled developmental stages of spermatogenesis in testis. Color indicates the different genotypes AA=red, AB*=green and B*B*=blue. Number indicates the testis development timepoint of the sample determined by histology as follows: 1=inactive, 2=accelerating I, 3=accelerating II, 4=active.

****A B**

Figure S4. Differential gene expression in testis and liver. A: Venn diagrams of overlapping DE genes between the tested contrast within the developmental timepoint pools in testis. Red indicates the upregulated and blue downregulated genes in each contrast. B: A: Venn diagram of overlapping DE genes between the tested contrast within liver. Red indicates the upregulated and blue downregulated genes in each contrast.


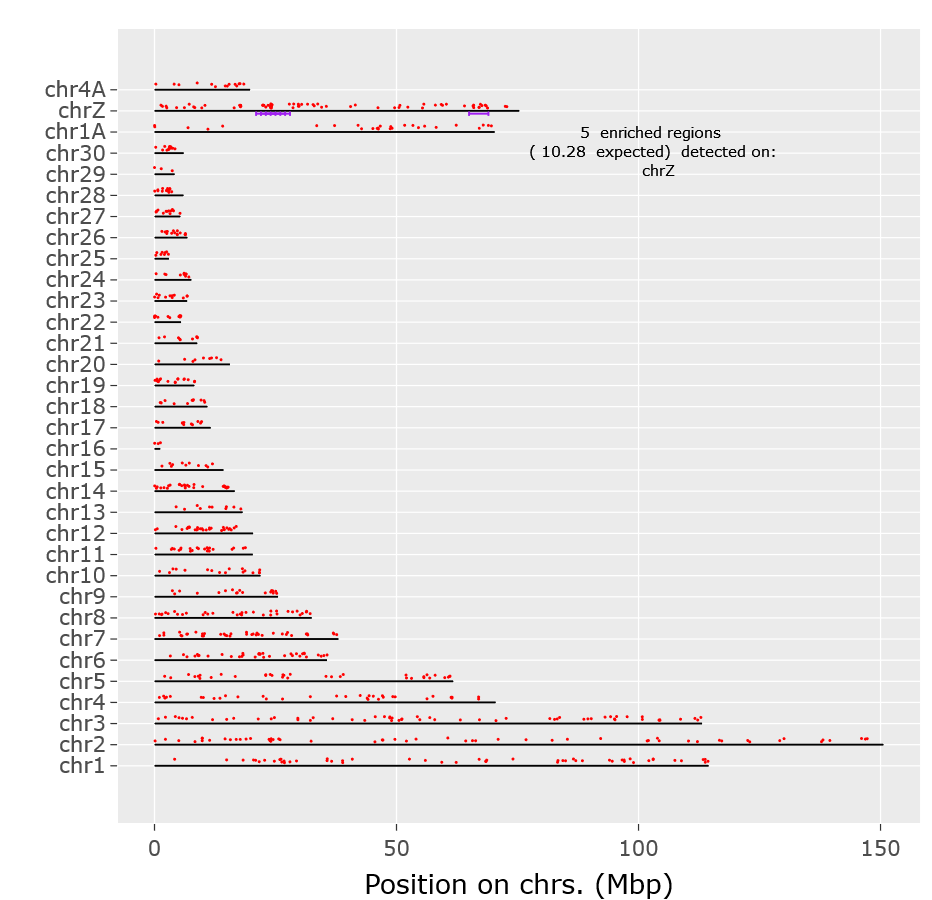


Figure S5. Overrepresentation of the 520 testis DE genes against the genomic background which includes all ethe 16 360 expressed genes from the transcriptome. Overrepresentation was determined with sliding window analysis using a window size of 4 Mb with 4 steps and with FDR cutoff of 0.01. Plot was produced with ShinyGO v0.77.


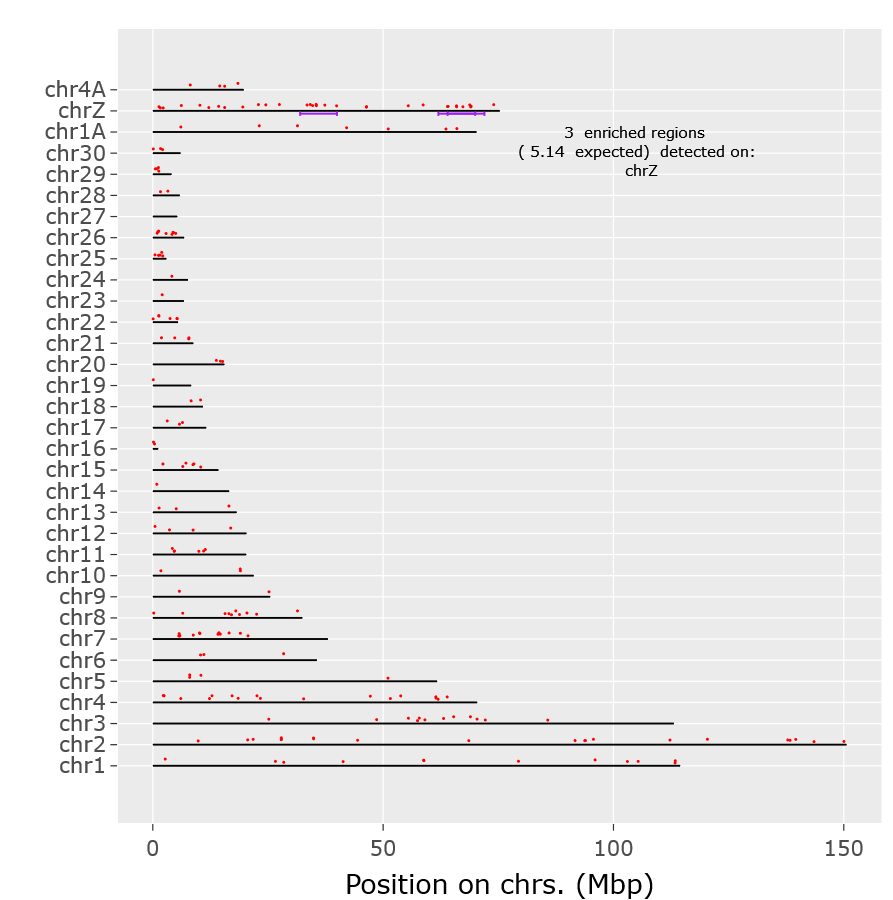


Figure S6. Overrepresentation of the 420 liver DE genes against the genomic background which includes all ethe 14 404 expressed genes from the transcriptome. Overrepresentation was determined with sliding window analysis using a window size of 8 Mb with 4 steps and with FDR cutoff of 0.01. Plot was produced with ShinyGO v0.77.


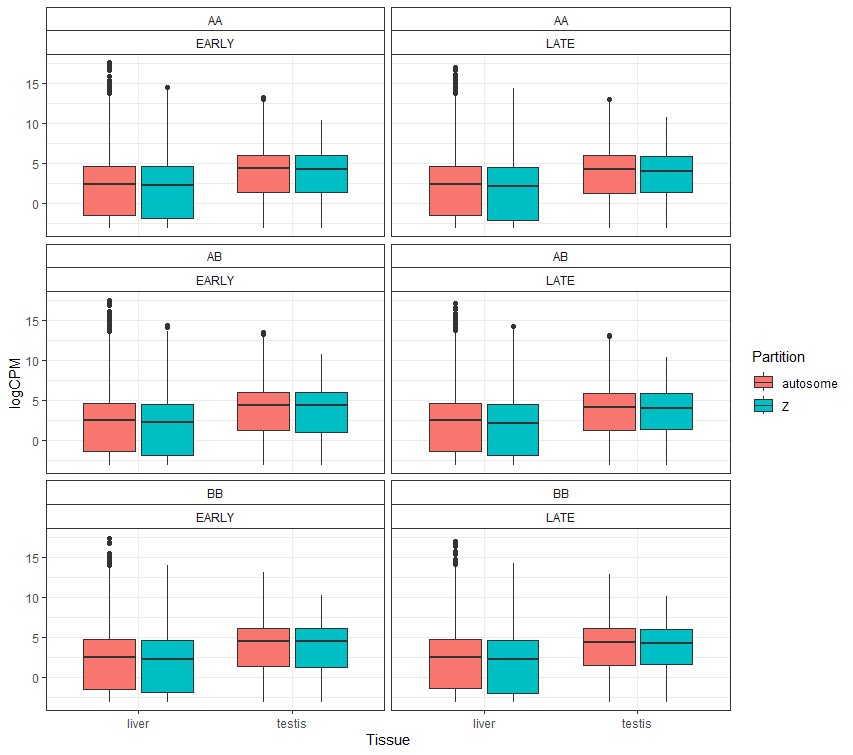


*Figure S7. Gene expression of Z chromosome genes and autosomal genes (scaffolds excluded) within the investigated developmental stages EARLY and LATE and karyotypes AA, AB* and B*B*. Expression level is logCP and liver is shown in red and testis tissue in blue*.


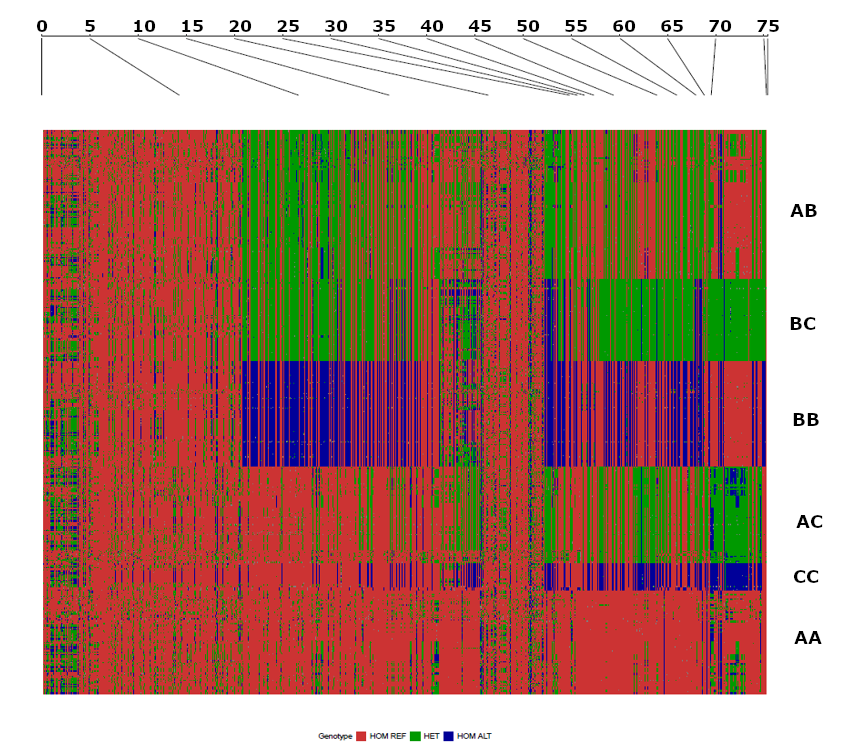


Figure S8. Kim et al (2017) SNP genotype data after rematch to taegut1.1. for chromosome Z. All samples are represented on vertical lines and at the top is represented the physical location of chromosome Z based on the location of the SNP. Each SNP genotype homozygous for the tagut1.1 reference is represented by red, homozygous for the alternative allele is in blue and heterozygote is in green. Top line represents physical location along chromosome Z based on the location of the SNP and downward line indicates the position among the SNPs as their density along the Z is not constant.


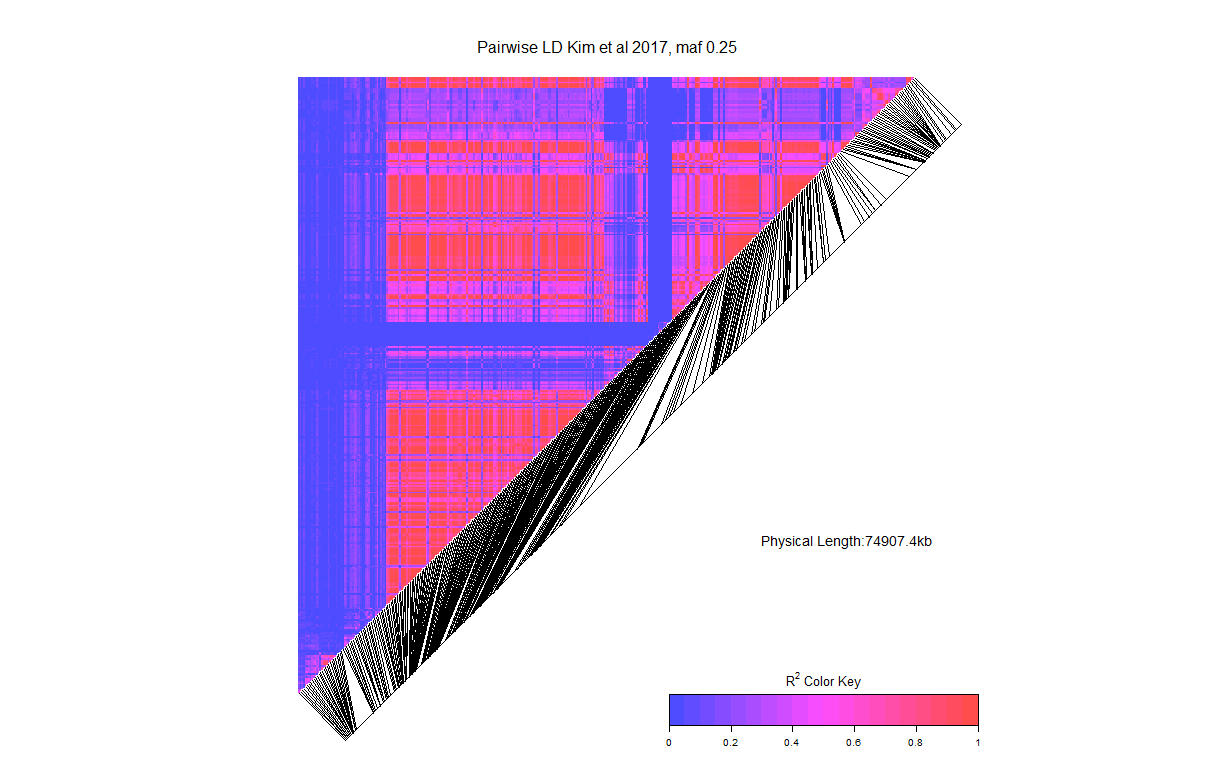

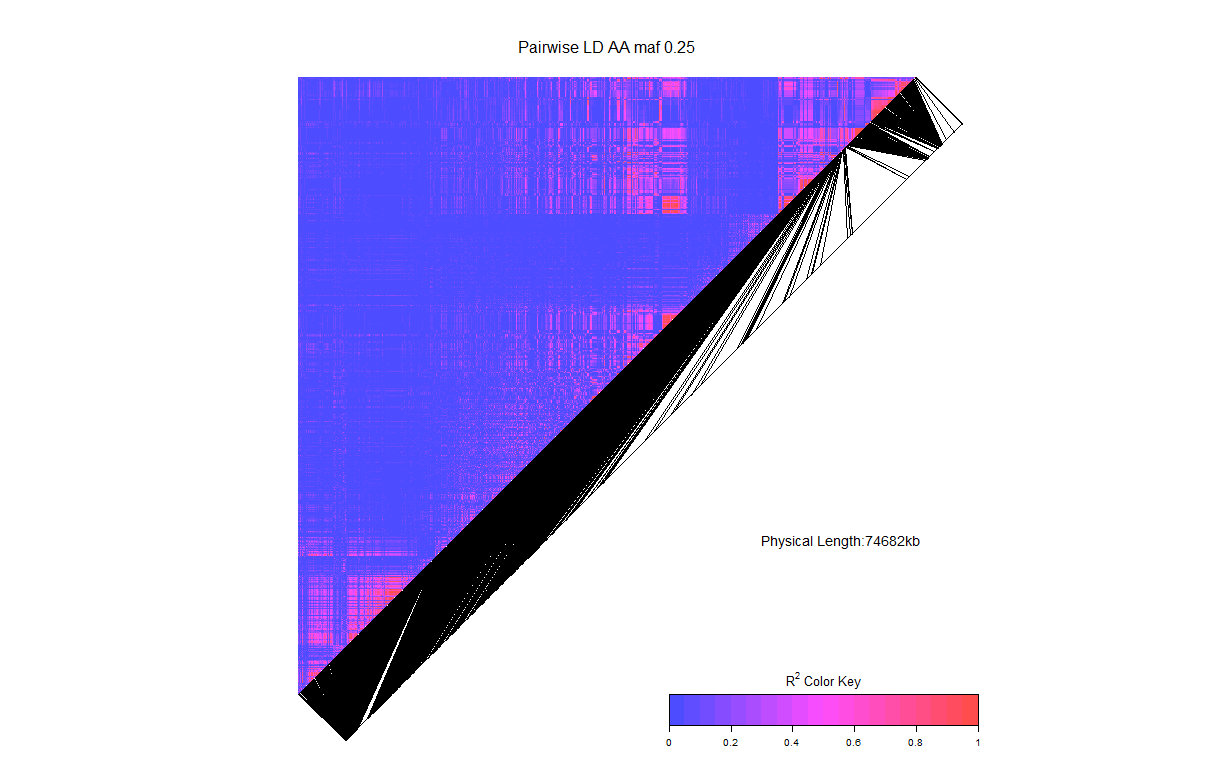

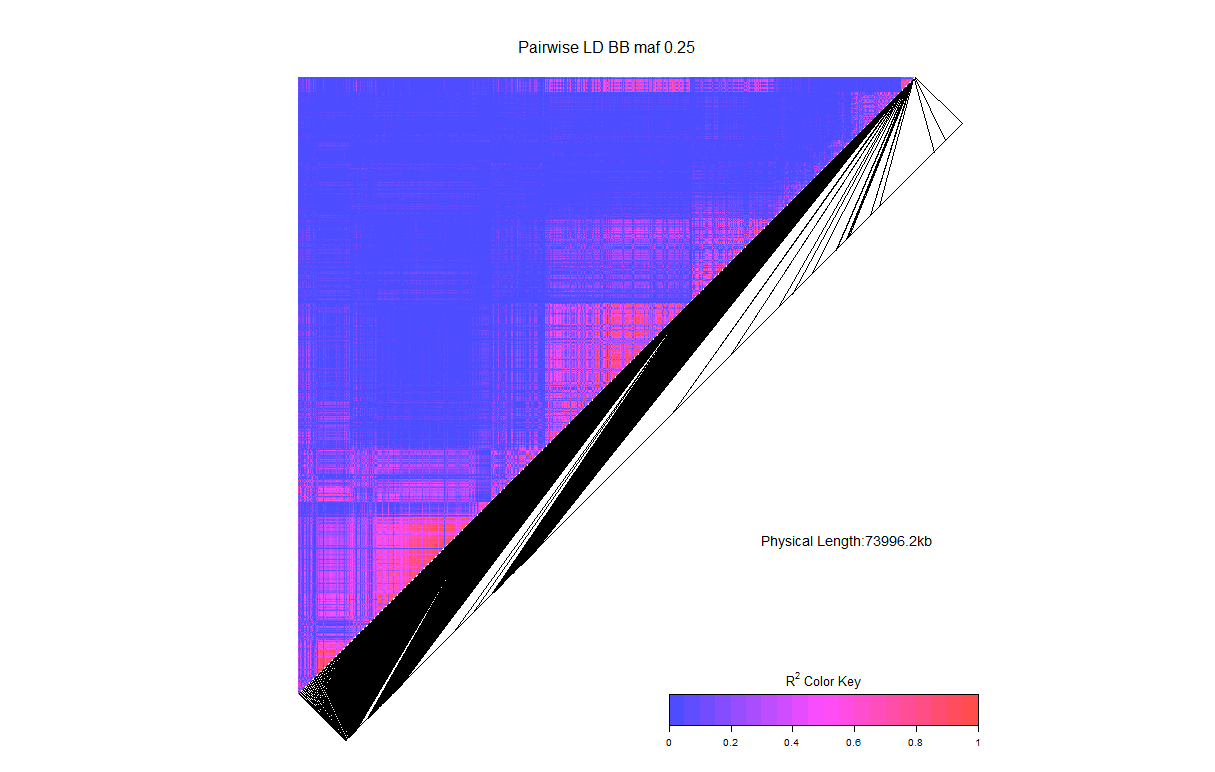

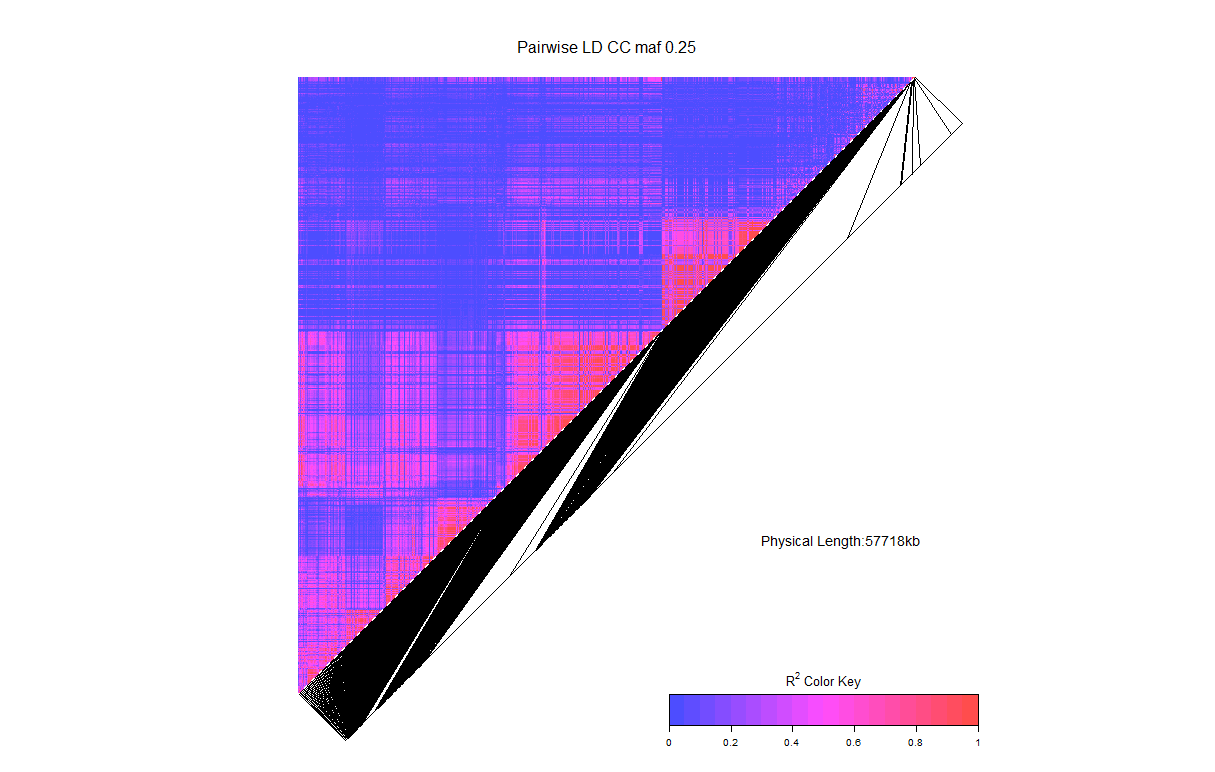


**D**

**B**

**C**

**A**

Figure S9. Pariwise LD plots within the SNP data from Kim et al. 2017 after rematch to taegut1.v1 and for the main inversion karyotypes from this data. A: full data thinned in plink to 378 SNPs. B: AA karyotypic individuals and 3333 SNPs. C: AA karyotypic individuals and 3333 SNPs. D: AA karyotypic individuals and 3333 SNPs.
